# A Risk-adapted Approach to Follow-up in Differentiated Thyroid Cancer

**DOI:** 10.5041/RMMJ.10231

**Published:** 2016-01-28

**Authors:** Valentina D. Tarasova, R. Michael Tuttle

**Affiliations:** Endocrinology Service, Memorial Sloan–Kettering Cancer Center, New York, NY, USA

**Keywords:** Clinical outcomes, follow-up, risk stratification, thyroid cancer

## Abstract

The last 10 years have seen a renewed interest in a risk-adapted approach to the management of differentiated thyroid cancer. This review outlines a state-of-the-art approach to individualized management in which the original follow-up plan that was developed based on initial risk stratification is modified over time as new data become available. This risk-adapted follow-up approach allows clinicians to determine the intensity of follow-up and management recommendations in response to real-time dynamic risk assessments which may change over time.

## INTRODUCTION

After completion of initial therapy, it is necessary to design a follow-up management strategy that will optimize the appropriate frequency and extent of additional testing that is required to identify persistent or recurrent disease in a timely fashion.^1^–^5^ However, an equally important goal is to identify the majority of thyroid cancer patients that go into remission in response to their initial therapy so that the intensity and frequency of their follow-up can be minimized since they are at very low risk of recurrence.

The cornerstone of this individualized follow-up management approach begins with appropriate initial risk stratification that defines each specific patient’s likelihood of remission, recurrence, distant metastasis, persistent disease, and need for additional therapy.^6^ Multiple organizations have validated and published initial risk stratification systems that can be used to guide initial management. While the American Joint Committee on Cancer (AJCC)/TNM and the MACIS scoring system can provide reasonably accurate predictions with regard to disease-specific mortality, other staging systems such as the American Thyroid Association (ATA) risk stratification system have been demonstrated to reflect the risk of disease recurrence, persistence, and remission more accurately.^4^,^5^,^7^

However, all of these initial risk stratification systems account for only about 15%–20% of the variability in the outcome that they are trying to predict.^6^ Therefore, it is necessary to modify these initial risk estimates over time as new data are accumulated during follow-up. These initial risk estimates may need to be changed either because the biologic behavior of the disease differs from what would have been predicted based on our assessment of the data available at diagnosis, or because the response to our initial therapies was either better or worse than we would have anticipated. Obviously, our initial risk assessments are only as good as the data we have available to us, implying that suboptimal imaging, pathology reports, surgical reports, and thyroglobulin assays will impair our ability to predict outcomes using any of these systems.^8^ From a clinical perspective, the concept of changing risk estimates over time is not new as we have always modified our treatment recommendations based on serum thyroglobulin, cross-sectional imaging, and FDG PET scan results, as well as radioactive iodine (RAI) scan results. For these reasons, multiple authors have now evaluated strategies to modify risk estimates over time and designated these systems either as response-to-therapy evaluation, delayed risk assessments, or dynamic risk assessments.^1^,^4^,^5^,^8^

**Figure 1.**
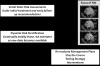
**Risk-adapted Approach to Management.**

In this review, we will evaluate how follow-up should be initially guided by our initial risk assessment using the ATA risk assessment model. We will then explore how the follow-up should be modified based on a response-to-therapy model incorporating new data into our risk assessment as it becomes available during follow-up (Figure 1). As described by one of our surgical colleagues, this approach allows the individual patient’s clinical picture to change from a fuzzy image at initial diagnosis to a sharp crisp picture as new data are obtained over time (personal communication, Michael Yeh, Endocrine Surgery, UCLA, Los Angeles, California, USA).

## USING INITIAL RISK ASSESSMENTS TO GUIDE INITIAL FOLLOW-UP

After completing initial therapy (thyroid surgery with or without RAI), it is necessary meticulously to gather all of the pertinent data available for each patient including all of the information obtained prior to surgery, as well as the intraoperative and postoperative findings, as it provides essential components for the ATA risk stratification. As noted by the ATA surgical affairs committee position paper, effective communication between all members of the disease management team is a requirement for optimal patient care.^9^

Using the modified 2009 ATA risk stratification system, patients can be classified as low, intermediate, or high risk (Table 1).^2^ A non-stimulated serum thyroglobulin value obtained about 6 weeks after surgery is also considered in the initial risk stratification system.^2^ A non-stimulated thyroglobulin above 5 ng/mL more than 6 weeks after a completion thyroidectomy would signal the clinician to look for the presence of residual normal thyroid tissue or metastatic disease that remains after surgery. These initial static risk assessments are used to plan the frequency and intensity of follow-up for approximately the first year, recognizing that the plan will likely change as new data become available.^6^

**Table 1.** ATA 2009 Risk Stratification System with Proposed Modifications.^2^

**Risk Level**	**Clinico-pathological Features**
ATA Low Risk	Papillary Thyroid Cancer (with all of the following): No local or distant metastases All macroscopic tumor has been resected No tumor invasion of loco-regional tissues or structures The tumor does not have aggressive histology (e.g. tall cell, hobnail variant, columnar cell carcinoma) If ^131^I is given, there are no RAI avid metastatic foci outside the thyroid bed on the first post-treatment whole-body RAI scan No vascular invasion Clinical N0 or ≤5 pathologic N1 micrometastases (<0.2 cm in largest dimension)^*^ Intrathyroidal, encapsulated follicular variant of papillary thyroid cancer^*^ Intrathyroidal, well-differentiated follicular thyroid cancer with capsular invasion and no or minimal (<4 foci) vascular invasion^*^ Intrathyroidal, papillary microcarcinoma, unifocal or multifocal, including V600E BRAF mutated (if known)^*^
ATA Intermediate Risk	Microscopic invasion of tumor into the perithyroidal soft tissues RAI avid metastatic foci in the neck on the first post-treatment whole-body RAI scan Aggressive histology (e.g. tall cell, hobnail variant, columnar cell carcinoma) Papillary thyroid cancer with vascular invasion Clinical N1 or >5 pathologic N1 with all involved lymph nodes <3 cm in largest dimension^*^ Multifocal papillary microcarcinoma with extrathyroidal extension and V600E BRAF mutated (if known)^*^
ATA High Risk	Macroscopic invasion of tumor into the perithyroidal soft tissues (gross extrathyroidal extension) Incomplete tumor resection Distant metastases Postoperative serum thyroglobulin suggestive of distant metastases Pathologic N1 with any metastatic lymph node ≥3 in largest dimension^*^ Follicular thyroid cancer with extensive vascular invasion (>4 foci of vascular invasion)^*^

Table from the ATA thyroid cancer guidelines.^2^ Used with permission from the American Thyroid Association.

* Proposed modifications, not present in the original 2009 initial risk stratification system.

The initial follow-up plan for low-risk patients usually includes a follow-up visit 6–12 months after our initial risk assessment, with a target thyroid stimulating hormone (TSH) in the 0.5–1.5 mIU/L range. In most patients, an ultrasound of the neck is planned in conjunction with the 12-month visit, although the utility of this test is unproven and could lead to finding more false-positive results than true disease if the serum thyroglobulin is undetectable during initial follow-up. Diagnostic RAI scans are seldom needed in the follow-up of low-risk patients because the diagnostic yield is low and nearly all recurrences in these patients can be identified by serum thyroglobulin (Tg) and neck ultrasonography. The primary goal of early follow-up for these patients is to identify those patients that demonstrate an excellent response to therapy (remission) who can quickly be transitioned to a much less intense follow-up program.

Intermediate-risk patients are initially followed at 6-month intervals with a target TSH in the 0.1–0.5 mIU/L range. In most cases, a neck ultrasound would be carried out at the 6-month time point (with another ultrasound done at the 1-year time point if there was extensive metastatic lymph node involvement present at the initial evaluation). Additionally, CT of the neck with contrast is considered at the 6–12-month follow-up in patients with initially extensive lateral neck metastatic lymphadenopathy to evaluate the neck region such as the retropharyngeal area that may not be adequately visualized with neck ultrasonography. Diagnostic RAI scans are not routinely obtained as a screen for recurrent/persistent disease. However, RAI scans may be used to characterize the functional status of structural disease identified during follow-up or to localize the source of markedly elevated or rising serum Tg levels. Additional testing is not planned, but may be necessary depending on the results obtained over the course of this first year as will be described below. The goal of follow-up for the intermediate-risk patients is to identify the 30% of these patients that will quickly go into remission and can be transitioned to less intense follow-up and the 70% of patients that do not go into remission and may benefit from additional observation, imaging, or intervention.

High-risk patients require very individualized follow-up plans which will be more dynamic than most intermediate- and low-risk patients. The majority of high-risk patients will have presumed or documented persistent disease after initial therapy. Therefore, the intensity and types of imaging used over the course of the first year will vary from case to case. However, in general, most high-risk patients will get their initial repeat imaging done 2–3 months after initial therapy. This usually includes imaging of the neck and chest as well as cross-sectional imaging of any known disease remaining after initial therapy. Since the radioactive iodine takes months to produce improvements in structural disease, these early scans are used more to identify patients that are failing initial therapy (having early disease progression) than to determine the effectiveness of our initial therapies. Recommendations for the rest of the first-year follow-up are largely driven by the findings of these 2–3-month imaging results and cannot be generalized effectively. Diagnostic RAI scans are frequently used in the follow-up of these patients, particularly if they had RAI avid disease identified as part of their initial therapy. The primary goal of early follow-up in the high-risk patients is to assess the response to initial therapy accurately and understand the underlying biologic behavior of their tumor. Over the first several months, the clinical course can be defined with much more confidence. Unless a patient’s co-morbid conditions provide a contraindication, the target TSH in high risk patients is usually 0.1 mIU/L.

## ASSESSING RESPONSE TO THERAPY

While the initial risk stratification allows the clinician to chart an initial follow-up course with a reasonable degree of confidence, it is well recognized that individual outcomes may differ from the predicted based on the initial prognostic features.^3^,^10^ In order to provide clarity and improve communication, the ATA guidelines endorsed a standardized and validated nomenclature that can be used to describe the clinical status of the patient at the time of each follow-up visit.^2^,^4^,^5^

**Figure 2.**
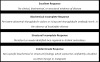
**Response-to-therapy Definitions.**

As can be seen in Figure 2, patients classified as having an excellent response to therapy have no biochemical, structural, or functional evidence of disease. A biochemical incomplete response to therapy identifies the patient with an abnormal serum thyroglobulin (either suppressed or stimulated) without structurally identifiable disease. The definition of biochemical incomplete response does not include specific serum thyroglobulin values because the definitions of what would be considered abnormal will vary based on initial therapy such as the extent of initial thyroid surgery and whether or not radioactive iodine ablation was done (discussed further below).

Patients with physical examination or imaging findings that are either biopsy-proven or highly suspicious for the presence of structurally identifiable disease are classified as having a structural incomplete response to therapy. The structural incomplete category does not differentiate persistent disease (present since the time of initial therapy) from recurrent disease (identified after a patient has previously been classified as having an excellent response to therapy) as the management decisions are based more on the presence or absence of structural disease than whether or not it is persistent or recurrent. In either case, it is the location and the rate of progression of the structural disease that guides the management, not whether it was classified as persistent or recurrent.

Finally, patients who have non-specific findings on imaging that cannot confidently be classified as having either excellent or structural incomplete response can be classified as having an indeterminate response to therapy which would lead to careful observation in most cases. Likewise, patients with serum thyroglobulin values in the very low range that do not meet the definition for excellent response, but are not high enough for biochemical incomplete response, are also classified as having an indeterminate response. As additional data are obtained over time, most patients with an indeterminate response will be reclassified into one of the other three response outcome categories, with only about 20% eventually being classified as truly having persistent disease.

Initial risk stratification can be very challenging (and probably less accurate) if the serum Tg values cannot be reliably measured (e.g. the presence of interfering anti-Tg antibodies) or if the tumor is a poor Tg producer (poorly differentiated cancers). Therefore, most patients with anti-Tg antibodies will be classified as indeterminate unless the titers are consistently rising over time, in which case they are categorized as having a biochemical incomplete response. Many of the poor Tg producers are relatively aggressive tumors and as such are classified as having indeterminate or structural incomplete response to therapy. However, in the absence of known structural disease, it is possible for a poor Tg producer to be erroneously classified as having an “excellent response.” Therefore, cross-sectional imaging (either CT or MRI) of at least the head and neck regions should be strongly considered before classifying a potentially poor Tg producer as having an excellent response to therapy.

One of the most important aspects of this response-to-therapy system is that, unlike AJCC/TNM staging, the MACIS score, or the ATA initial risk stratification system, it is dynamic and may change over time for an individual patient as new data are accumulated.^6^ For example, it is very common for a patient with a biochemical incomplete response to have serum thyroglobulin values gradually decline over time and be reclassified as having an excellent response.^7^ Conversely, a small number of patients that initially demonstrate an excellent response will develop abnormal thyroglobulin values over time or have small lymph node metastasis identified during follow-up, in which case they may be reclassified as having either a biochemical or structural incomplete response at the time of the follow-up visit. This dynamic nature of the system allows clinicians to recognize when the clinical course is changing for a particular patient and to adapt the management accordingly.

## CLINICAL OUTCOMES BASED ON RESPONSE-TO-THERAPY ASSESSMENTS IN PATIENTS TREATED WITH TOTAL THYROIDECTOMY AND RADIOACTIVE IODINE (RAI) ABLATION

**Figure 3.**
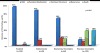
**Risk Estimates Using Response-to-therapy Assessment.** Total thyroidectomy and radioactive iodine remnant ablation (*n*=471, MSKCC, median follow-up 7 y).

As shown in Figure 3, the clinical outcomes vary dramatically based on the best response to initial therapy in a cohort of 471 differentiated thyroid cancer patients followed for a median of 7 years at Memorial Sloan–Kettering Cancer Center (MSKCC) (Figure 3).^4^,^5^ In this cohort, 96% of the patients that achieved an excellent response to therapy continued to have no evidence of disease at final follow-up. Likewise, the majority of patients (87%) with an indeterminate response also had no disease at final follow-up. Interestingly, two-thirds of the patients with a biochemical incomplete response to initial therapy had no evidence of disease at final follow-up, with the remainder demonstrating either biochemical or structural evidence of disease at the final follow-up. It is important to note that while an excellent response to therapy predicts a risk of recurrence of 1%–2% in ATA low- and intermediate-risk patients, the few patients presenting with ATA high risk that subsequently demonstrate an excellent response still have a substantial risk of recurrence (5%–15%) and require more vigilant follow-up. Patients that demonstrated a structural incomplete response to therapy did far worse than the other three groups. Despite additional treatments, very few of these patients could be classified as having no evidence of disease. Most of these patients had persistent biochemical or structural evidence of disease, and all of the patients that died of thyroid cancer demonstrated an initial structural incomplete response to therapy.

The ATA guidelines reviewed the numerous publications examining the clinical outcomes and management implications of the response-to-therapy outcome categories in great detail (Table 2).^2^ Consistently with the finding of the original Memorial Sloan–Kettering Cancer Center cohort,^4^,^5^ multiple studies now confirm a median recurrence rate of about 1.5% in patients that have an excellent response to initial therapy. The majority of the patients with a biochemical incomplete response are classified as having no evidence of disease at final follow-up, either spontaneously or after additional therapy. There were no disease-specific deaths in this cohort, and only 20% developed structural disease, almost always associated with rising thyroglobulin values over time. Similarly, the indeterminate response category developed structurally identifiable disease, and only 15%–20% of the remainder had non-specific changes that were either stable or resolved over time. The poorest outcomes are routinely seen with the structural incomplete response category, with the vast majority of these patients having persistent disease at final follow-up, and with disease-specific death rates as high as 11% if the structural incomplete response is associated with disease in the neck and 50% in patients with distant metastases.^5^

**Table 2.** Clinical Implications of Response-to-therapy Reclassification in Differentiated Thyroid Cancer Patients Treated with Total Thyroidectomy and RAI Remnant Ablation.

**Category**	**Definitions**	**Clinical Outcomes**	**Management Implications**
Excellent Response	Negative imaging *AND either* Suppressed Tg <0.2 ng/mL^*^ or TSH-stimulated Tg <1 ng/mL^*^	1%–4% recurrence <1% disease-specific death	An excellent response to therapy should lead to an early decrease in the intensity and frequency of follow-up and the degree of TSH suppression
Biochemical Incomplete Response	Negative imaging *AND* Suppressed Tg >1 ng/mL^*^ or Stimulated Tg >10 ng/mL^*^ or Rising Tg Ab levels	At least 30% spontaneously evolve to NED 20% achieve NED after additional therapy 20% develop structural disease <1% disease-specific death	If associated with stable or declining serum Tg values, a biochemical incomplete response should lead to continued observation with ongoing TSH suppression in most patients. Rising Tg or Tg antibody values should prompt additional investigations and potentially additional therapies
Structural Incomplete Response	Structural or functional evidence of disease With any Tg level +/− Tg Ab	50%–85% continue to have persistent disease despite additional therapy Disease-specific death rates as high as 11% with loco-regional metastases and 50% with structural distant metastases	A structural incomplete response may lead to additional treatments or ongoing observation depending on multiple clinico-pathologic factors including the size, location, rate of growth, RAI avidity, ^18^FDG avidity, and specific pathology of the structural lesions
Indeterminate Response	Non-specific findings on imaging studies Faint uptake in thyroid bed on RAI scanning Non-stimulated Tg detectable, but less than 1 ng/mL Stimulated Tg detectable, but less than 10 ng/mL or Tg antibodies stable or declining in the absence of structural or functional disease	15%–20% will have structural disease identified during follow-up In the remainder, the non-specific changes are either stable, or they resolve <1% disease-specific death	An indeterminate response should lead to continued observation with appropriate serial imaging of the non-specific lesions and serum Tg monitoring. Non-specific findings that become suspicious over time can be further evaluated with additional imaging or biopsy

Table from the ATA thyroid cancer guidelines.^2^ Used with permission from the American Thyroid Association.

* In the absence of Tg antibodies (Tg Ab).

NED, no evidence of disease at final follow-up.

## MANAGEMENT IMPLICATIONS OF THE RESPONSE-TO-THERAPY CATEGORIES IN PATIENTS TREATED WITH TOTAL THYROIDECTOMY AND RAI ABLATION

As would be expected, the response-to-therapy outcome categories can be used to guide clinical management recommendations (Table 2).^2^ Given the very low risk of recurrence in patients who achieved an excellent response to initial therapy, a minimalistic approach to follow-up should be employed. Patients demonstrating an excellent response to therapy can be followed on a yearly basis and managed primarily with physical exam and serum thyroglobulin values every 1–2 years. There is little role for neck ultrasound or cross-sectional imaging on a routine basis for these patients as the risk of false negatives almost certainly exceeds the likelihood of finding structural disease. There is no role for TSH suppression in this group (TSH target approximately 0.5–2.0 mIU/L).

In patients with a biochemical incomplete response, TSH is usually maintained in the 0.5–1.0 mIU/L range, with the time and intensity of cross-sectional and functional imaging determined by the trend in the suppressed serum thyroglobulin. In patients with a declining serum thyroglobulin over time, observation with an occasional neck ultrasound every 2–3 years is reasonable. However, if serum thyroglobulin is increasing, then additional imaging would be warranted based on the magnitude of the thyroglobulin level and the rate of rise over time, often expressed as the thyroglobulin doubling time.^11^

An indeterminate response usually just mandates serial observation in order to follow the mildly abnormal thyroglobulin or non-specific changes on the cross-sectional imaging over time so that the patient can eventually be reclassified as having either excellent, or biochemical or structural incomplete response. Similarly to a biochemical incomplete response, TSH is usually maintained in the 0.5–1.0 mIU/L range in this group of patients.

The most challenging group is the patients with the structural incomplete response. Depending on the location of the disease, the rate of structural progression, the RAI avidity, the magnitude of FDG PET avidity, and the response to previous therapies, management options can vary from observation, to surgical intervention, to external beam radiation, to systemic therapies. Decision-making in patients with a structural incomplete response to therapy requires the clinician and the patient carefully to weigh the risk and benefit of additional therapy as the majority of the patients will not be rendered disease-free even with additional treatments. Unless contraindicated by other medical conditions, the TSH target for patients with structural incomplete response is usually about 0.1 mIU/L.

Finally, it is important to emphasize that the response-to-therapy categories need to be interpreted in light of the initial risk estimates. This is most clearly evident in patients that demonstrated an excellent response to therapy and who were initially classified as high-risk patients. Despite having an excellent response to therapy, their risk of recurrence may be as high as 5%–15% depending on their high-risk features^6^ as opposed to the 1%–2% risk of recurrence seen in patients with an excellent response who were initially classified as low risk. Clinicians need to be aware that some of these high-risk patients can be inappropriately classified as having an excellent response to therapy because they are poor thyroglobulin producers or because they have metastatic sites that are not readily apparent on our standard initial imaging.

## CLINICAL OUTCOMES BASED ON RESPONSE-TO-THERAPY ASSESSMENTS IN PATIENTS TREATED WITH LESS THAN TOTAL THYROIDECTOMY AND RAI ABLATION

While most of the work on response to therapy is focused on patients that had total thyroidectomy and RAI ablation, these same response-to-therapy categories can also be applied to patients treated with either lobectomy or total thyroidectomy without RAI.^6^ The only modification required is to change the serum Tg cut-points that define excellent, indeterminate, and biochemical incomplete response. Recently, we proposed and validated specific serum thyroglobulin cut-point values that allow classification of the response to therapy in these patients as well (Table 3).^6^

**Table 3.** Serum Thyroglobulin Values that Define Response-to-therapy Categories Based on Initial Treatment Strategy.

	**Total Thyroidectomy and RAI Ablation**	**Total Thyroidectomy**	**Lobectomy**
Excellent	<0.2 ng/mL	<0.2 ng/mL	<30 ng/mL
Indeterminate	0.2–1.0 ng/mL	0.2–5.0 ng/mL	–
Biochemical Incomplete	>1.0 ng/mL	>5.0 ng/mL	≥30 ng/mL

By definition, patients appropriately selected for thyroid lobectomy have low-risk tumors and are expected to have low recurrence rates and essentially no disease-specific mortality. Patients treated with thyroidectomy without RAI ablation can either be in low- or intermediate-risk groups demonstrating structural recurrence rates of approximately 5%. In patients who did not receive RAI therapy, neck ultrasound coupled with the trend in thyroglobulin values over time has the most value in identifying recurrent thyroid cancer. The TSH target for a low-risk patient selected for either lobectomy or total thyroidectomy without RAI ablation is usually in the 0.5–2.5 mIU/L range and does not require exogenous levothyroxine if the normal contralateral lobe functions adequately. Similarly, patients demonstrating an excellent response to therapy do not require TSH suppression while those classified as having an indeterminate or biochemical incomplete response may benefit from mild suppression with TSH in the range of 0.5–1.0 mIU/L.

Our primary follow-up for low-risk patients treated with lobectomy or total thyroidectomy without RAI includes a neck ultrasound 6–12 months after initial therapy with a non-stimulated serum thyroglobulin and thyroglobulin antibodies done at the same time. If these studies are unremarkable, serum thyroglobulin values are obtained yearly, with ultrasound at 3–5 year intervals, and with a TSH maintained in the 0.5–2.5 mIU/L range.

## CONCLUSIONS

State-of-the-art management of differentiated thyroid cancer must include a risk-adapted approach to the initial therapy and ongoing dynamic assessment of the response to therapy during follow-up in order to optimize individualized patient care. Following initial therapy, early management recommendations should be based on individualized estimates of the risk of recurrence and the risk of disease-specific mortality using the ATA risk stratification system and the AJCC/TNM staging system. The initial risk assessment determines the initial management strategy, balancing the risks of recurrence with the benefits of early disease detection, and tailors a plan that optimizes detection of clinically significant disease while minimizing excessive testing and the risks of false-positive findings. A response-to-therapy assessment model is then used to modify these initial risk estimates as new clinical data become available and to guide further management in a risk-adapted fashion for each patient. This strategy optimizes individualization of care by providing a continual re-evaluation of both the clinical status of each patient and the follow-up plan at each encounter.

## Abbreviations:

AJCC: American Joint Committee on Cancer
ATA: American Thyroid Association
RAI: radioactive iodine
Tg: thyroglobulin
TSH: thyroid stimulating hormone.
